# Influence of single and binary doping of strontium and lithium on *in vivo* biological properties of bioactive glass scaffolds

**DOI:** 10.1038/srep32964

**Published:** 2016-09-08

**Authors:** Pintu Kumar Khan, Arnab Mahato, Biswanath Kundu, Samit K. Nandi, Prasenjit Mukherjee, Someswar Datta, Soumya Sarkar, Jayanta Mukherjee, Shalini Nath, Vamsi K. Balla, Chitra Mandal

**Affiliations:** 1 Department of Veterinary Surgery and Radiology, West Bengal University of Animal and Fishery Sciences, Kolkata, India; 2 Bioceramics and Coating Division, CSIR-Central Glass and Ceramic Research Institute, Kolkata, India; 3 Non-Oxide Ceramics and Composites Division, CSIR-Central Glass and Ceramic Research Institute, Kolkata, India; 4 Institute of Animal Health and Veterinary Biologicals, Animal Resources Development Department, Kolkata, India; 5 Cancer Biology and Inflammatory Disorder Division, CSIR-Indian Institute of Chemical Biology, Kolkata, India

## Abstract

Effects of strontium and lithium ion doping on the biological properties of bioactive glass (BAG) porous scaffolds have been checked *in vitro* and *in vivo*. BAG scaffolds were prepared by conventional glass melting route and subsequently, scaffolds were produced by evaporation of fugitive pore formers. After thorough physico-chemical and *in vitro* cell characterization, scaffolds were used for pre-clinical study. Soft and hard tissue formation in a rabbit femoral defect model after 2 and 4 months, were assessed using different tools. Histological observations showed excellent osseous tissue formation in Sr and Li + Sr scaffolds and moderate bone regeneration in Li scaffolds. Fluorochrome labeling studies showed wide regions of new bone formation in Sr and Li + Sr doped samples as compared to Li doped samples. SEM revealed abundant collagenous network and minimal or no interfacial gap between bone and implant in Sr and Li + Sr doped samples compared to Li doped samples. Micro CT of Li + Sr samples showed highest degree of peripheral cancellous tissue formation on periphery and cortical tissues inside implanted samples and vascularity among four compositions. Our findings suggest that addition of Sr and/or Li alters physico-chemical properties of BAG and promotes early stage *in vivo* osseointegration and bone remodeling that may offer new insight in bone tissue engineering.

The management of bone defects still remains a challenge for orthopedic surgeons. It has been reported that occurrence of impaired fracture healing of bone defects is nearly 5–10%^1^. In United States alone, 1.3 million people undergo bone graft surgeries each year for skeletal defects resulting from either accidents or disease^2^. Bone grafting, either from autografts or allografts is a well-known surgical procedure although has associated drawbacks of additional surgery, limited availability, potential risks of disease transmission, immunogenic response and long-term complications^3^. To overcome these limitations, the development as well as the availability of new orthobiologic materials to aid in the management of bony defects is rising.

On the other hand, tissue engineering mainly with engineered grafts is now-a-days a major thrust area toward repair and replacement of these diseased and damaged bone tissues. To achieve this goal, bioactive glasses and calcium phosphate have been investigated as bone repair scaffolds but having some limitations^4^,^5^. Mechanical and osteoinductive properties of scaffold materials can be improved via metallic ions substitution^6^. Among various ion substitutions, strontium (Sr^2+^), zinc (Zn^2+^), magnesium (Mg^2+^) and silicon (Si^4+^) have been widely studied^7^,^8^,^9^,^10^,^11^,^12^,^13^. Sr was the only one that was correlated with an increase in bone compression strength^14^. Stimulatory effect of Sr on osteoblasts and inhibitory effect on osteoclasts have been established^15^,^16^,^17^,^18^. Further, strontium ranelate has been proven to reduce the incidence of fractures in osteoporotic elderly patients^19^. Similarly, lithium (Li^+^) plays a vital role in osteoblast proliferation and differentiation through stimulation of the Wnt signaling pathway^20^,^21^. An earlier study demonstrated increased *in vitro* proliferative activity only at low Li^+^ concentration (0.25 wt. %)^22^.

Bioactive glasses with interconnected porosity with large surface areas are also favorable for bone integration. These materials with such structures have also been found to support angiogenesis^23^,^24^, osteoproduction by stimulating proliferation and differentiation of osteoprogenitor cells through direct genetic control^25^. Glass-ceramics and bioactive glasses with Sr^2+^ doping have been attempted recently^26^,^27^. Strontium-doped bioactive glasses have also been developed^28^.

Single and binary doping of Sr^2+^ and Li^+^ on *in vivo* bone regeneration of bioactive glasses scaffolds has not been reported yet. In the present study we have made an attempt to assess the beneficial effects of Sr^2+^ and Li^+^ doping on *in vivo* bone formation of an interconnected bioactive glass porous scaffold developed in the laboratory through rabbit bone defect model. Detailed phase, composition and microstructure analysis were performed prior using tools like X-ray diffraction (XRD), Fourier transformed infrared spectroscopy (FTIR), differential thermal analysis-thermo-gravimetric analysis (DTA-TGA), quantitative EDAX analysis and scanning electron microscopy (SEM) respectively. The scaffolds were also assessed for its bioactivity in contact with simulated body fluid (SBF) and *in vitro* cyto-toxicity by MTT assay using NIH3T3. The *in vivo* bone regeneration was analyzed using chronological radiography, fluorochrome labeling, SEM, histology and micro-computed tomography (μ-CT).

## Materials and Methods

### Bioactive glass scaffold preparation

Bioactive glass (with or without doping of Li or Sr) was prepared through conventional glass melting procedure using appropriate amounts of reagents like silica (SiO_2_), calcium carbonate (CaCO_3_), dry soda ash (Na_2_CO_3_), decahydrated borax (Na_2_B_4_O_7_.10H_2_O), TiO_2_, di-ammonium hydrogen ortho-phosphate, lithium and strontium carbonate (all inorganic chemicals including the ones referred later in the manuscript were analytical grade from M/s S.D. Fine-Chem Limited, India until specified seperately). Briefly, reagents were first mixed homogeneously, melted at 1450 °C in a Pt-crucible, homogeneity was maintained while melting and finally quenched in water to obtain the cullet. Dried cullets were further milled in a high energy ball mill for 3 h in aqueous medium. The final compostion of the as-prepared glass powders (obtained by ICP-AES chemical analysis) is given in Table 1. 0.25% Li_2_O and 1% SrO (by weight) doping was used strategically for the base glass composition and nomenclatures like BAG, L-BAG, S-BAG and LS-BAG were given for bioactive glass without doping, Li-, Sr- and binary Li + Sr substitution respectively and used throughout the manuscript.

To fabricate porous scaffolds, milled as-prepared respective glass powders were first mixed with an equal quantity of porogen (scintillation grade naphthalene). The resultant mix was compacted at 150 MPa in a cold-isostatic press (EPSI, Belgium); cut into 8 mm diameter specimens using a low speed diamond saw (Isomet, Buehler, USA). Subsequently, naphthalene was driven off very slowly (from r.t. to 80 °C) with pre-determined rate of schedule, followed by heat treatment at 675 °C except for LS-BAG (650 °C) on a Pt-plate for 6 min. These temperatures were selected after careful assessment of glass-transition temperatures (T_g_) mentioned later. The samples were finally stored in a vacuum desiccator until further use.

### Bioactive glass powders and scaffold characterization

Both as-prepared and heat treated powders were analyzed for phase by XRD at a diffraction angle of 10–80°2θ [X’Pert Pro, Phillips Analytical, Netherlands; Cu K_α1_ radiation; scan speed 2° min^−1^. FTIR transmittance spectra was recorded at mid-IR range (4000–400 cm^−1^) [Spectrum 100, PerkinElmer, USA; resolution: 2 cm^−1^; by KBr pellet method] to confirm the functional groups present. On the other hand, DTA-TGA was conducted to determine the thermal profiles of the glass powders [STA 449C, Netzsch, Germany; rate of heating upto 1000 °C: 10 °C/min. with initial sample mass of 5 ± 0.5 mg]. Heat treatment temperatures of BAG, L-BAG, S-BAG and LS-BAG were selected by assessing the Tg by the same DTA-TGA analysis.

Further, porous scaffolds were first physically characterized for open or apparent porosity (A.P.) and bulk density (B.D.) by water displacement method (Archimedes’ principle), then by a table-top SEM (Phenom pro-X, Netherlands) for detailed microstructural characterization and assessment of pore size, shape and morphology with Au/Pd sputter coating on the samples prior. B.D. was calculated by [D/(W-S)] and A.P. by [(W-D)/(W-S)] x 100%, where D, W and S are dry, soaked and suspended weight of the samples respectively while calculating by the methods mentioned. Variations of pore sizes were calculated by processing several SEM images thus obtained and using free software available (Perfect Screen Ruler v. 2.0) subsequently.

### SBF bioactivity study

Primary bioactivity study of the bare scaffolds was carried out in contact with SBF, before *in vitro* cell cytotoxicity and *in vivo* pre-clinical study just to check calcium and phosphate ion deposition ability of the samples. Supernatants were analyzed for Ca^2+^, HCO_3_^−^, and HPO_4_^−^ ions. SBF was prepared as per Kokubo *et al.*^29^. All the samples were selected with surface area of sample and volume of SBF taken with ratio of 2 mm^2^/mL (e.g., 40 mm^2^/20 mL) and soaked for 7 and 14 days. Samples were kept statically at a temperature 37.4 °C with pH 7.4 inside an incubator. After 7 and 14 days, ion concentrations were plotted and analyzed. One of respective samples (from BAG, L-BAG, S-BAG and LS-BAG) was seen for microstructure by FESEM (using Sigma, Carl Zeiss, Germany) after day 14. The samples were dried and carbon sputter coated prior observation.

### *In vitro* cell cytotoxicity

Chemicals used: Iscove’s modified Dulbecco’s medium (IMDM), phosphate-buffered saline (PBS), formaldehyde, 3-(4,5 dimethyl thiazol-2yl)-2,5 diphenyltetrazolium bromide (MTT), osmium tetroxide, paraformaldehyde, dimethyl sulfoxide (DMSO) were from Sigma Aldrich, St. Louis USA; fetal bovine serum (FBS) and penicillin/streptomycin, trypsin-ethylenediaminetetra acetic acid (EDTA), L-glutamine were from Invitrogen, CA,USA.

Fibroblast (NIH3T3) cell line (NCCS, Pune, India) was cultured in IMDM supplemented with 10% FBS and 1% penicillin-streptomycin (Complete Medium) in a humidified atmosphere of 5% CO_2_ at 37 °C. BAG, S-BAG, L-BAG, LS-BAG scaffolds were autoclaved and then rinsed with 70% alcohol for sterilization. NIH3T3 (2 × 10^3^) cells were seeded on scaffolds which were previously placed in a 24 well plate. Cells were allowed to attach on the surface of scaffolds for 2 h. Subsequently another 1.0 mL of complete medium was added in each well and cultured on for 3 and 7 days. Culture medium was changed in every 2 days.

To test cytotoxicity of BAG, S-BAG, L-BAG, LS-BAG scaffolds on cells, MTT assay was carried out after 3 and 7 days of culture. Cell without scaffold was used as control. MTT solution was prepared by dissolving MTT (5.0 mg) in DMSO (1.0 mL). MTT (100 μL) solution was diluted with IMDM (900 μL). After removing the previous medium diluted MTT solution was added in each well and incubated for 3 h. Purple coloured formazan crystal which are formed by the oxidation of tetrazolium salt by mitochondrial succinate dehydrogenase enzyme was dissolved in DMSO (500 μL). The resulting solution (200 μL) was placed into a 96 well plate and the optical density at 550 nm was measured using plate reader (Thermo Scientific). Each experiment was carried out in triplicate and the results were presented as means ± SD.

### Cell morphology by SEM

NIH3T3 (2 × 10^3^) cells were seeded on scaffolds and cultured for 7 days. Scaffolds containing cells were washed with PBS (0.1 M) and fixed with paraformaldehyde (2%) overnight at 4 °C. Subsequently, it was fixed with 2% osmium tetroxide (OsO_4_) for 2 h at 25 °C. The fixed samples were then dehydrated in an ethanol series 30%, 50%, 70%, 90% and 100% each for three times, followed by gold sputter coating for SEM observation of cell morphology.

### In vivo study of rabbit femoral bone defect model

**Figure 1:**
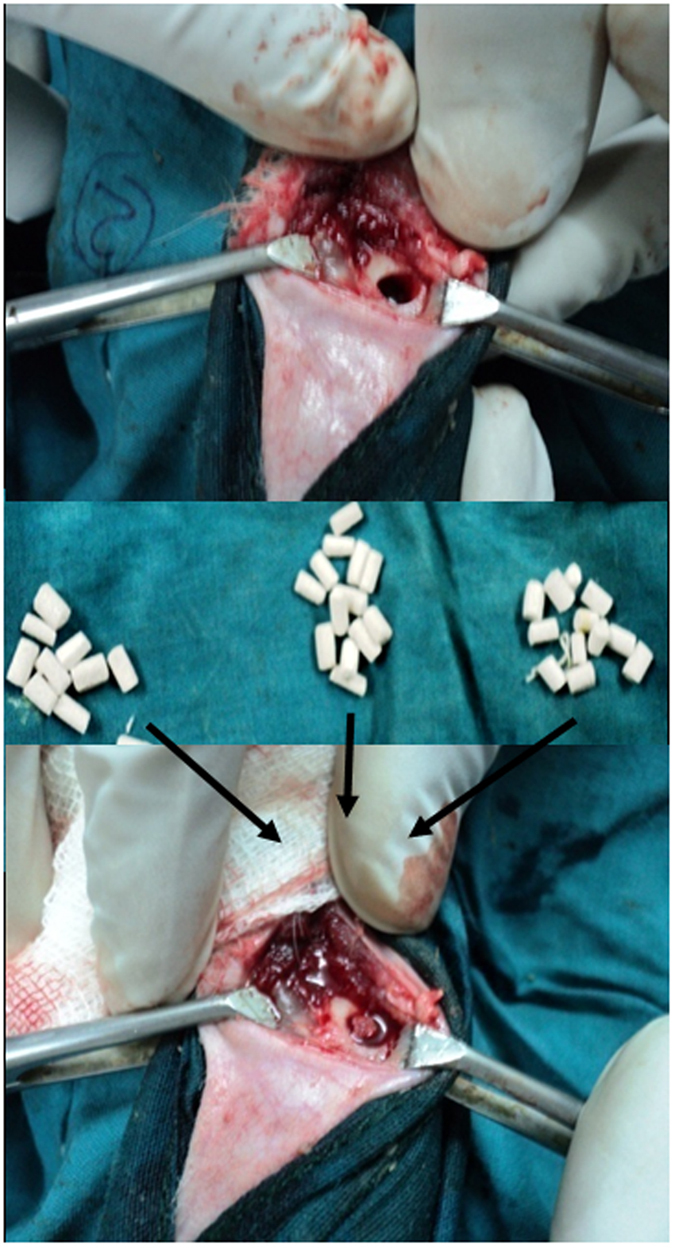
Surgical placement of the porous scaffolds (with or without doped BAG).

The animal experiments were performed following an ethical committee approved protocol in accordance with Institutional Animal Ethical Committee (IAEC), West Bengal University of Animal and Fishery Sciences (WBUAFS), West Bengal, India (Permit No. Pharma/IAEC/34 dated 30 June 2014). Sixteen adult New Zealand White rabbits (1.5–2 kg) were randomized into four groups (n = 4): control group I (pure BAG) and the test animals, group II (S-BAG), group III (L-BAG) and group IV (LS-BAG) with bilateral implantation. All surgeries were performed under general intramuscular anesthesia using xylazine hydrochloride (6 mg/kg) (Xylaxin, Indian Immunologicals, India) and ketamine hydrochloride (33 mg/kg) (Ketalar, Parke-Davis, India). Scaffolds were press fitted within the created defects in the distal metaphyseal region of femur and wounds were sutured in three layers (Fig. 1). Subsequently, animals were administered with cefotaxime sodium (Mapra India, India) at 20 mg/kg body weight intramuscularly for 5 days twice daily at 12 h interval and meloxicam at 0.2 mL (Intas Pharmaceuticals, India) once daily. Animals were finally sacrificed after 2 and 4 months of implantation.

### Characterization of *in vivo* samples

Bone healing in the defect was monitored using chronological radiographs taken immediately after implantation and once in a month up to 4 months. Radiographs were scored independently by double blinded investigators per methods described by Zhukauskas *et al.*^30^ (Table 2). For histological analysis, the bone specimens from the healed bone defect were collected, washed thoroughly with normal saline and was immediately fixed in 10% formalin for 7 days. Subsequently, the bone tissues were decalcified using Goodling and Stewart’s fluid containing 15 mL formic acid, 5 mL formalin and 80 mL distilled water, followed by fixation with 4% paraformaldehyde. Finally, the samples were embedded in paraffin wax and 4 μm sections were cut from the mount and stained with haematoxyline and eosin finally. Additional scoring system was developed from the histological slide using several *in vivo* biological activities (cellular response) and the response score was marked with ‘0’ for absence, ‘1’ for mild, ‘2’ for moderate, ‘3’ for marked and ‘4’ for severe activity.

For another set of samples, fluorochrome, i.e. oxytetracycline dehydrate (Pfizer India, India) was intramuscularly injected 3 weeks before sacrifice at two time points, i.e., 2 and 4 months (administered at 25 mg/kg body weight). Undecalcified ground sections (20 μm) were prepared from implanted segments of bone using different grades of sand paper and observed under UV light with Leica DM 2000 bright light phase contrast and fluorescence microscope including Leica Qwain software. Golden yellow fluorescing area was observed to identify newly formed bone and was also measured in μm^2^ and converted to percentage of bone formation. The extracted samples were also observed using SEM for bone-implant interfacial characteristics. Samples were first fixed in 5% glutaraldehyde in PBS buffer for 48 hours followed by gradual ethanol series drying. Dried samples were gold coated before imaging using the same desktop SEM, described earlier.

Micro-computed tomography (μ-CT) images of extracted bone samples with inserted scaffolds (BAG, L-BAG, S-BAG and LS-BAG) were taken using XT-H 225 (Nikon Metrology, Belgium) with maximum 110 kV rotating target X-ray source (75 μA test current), spot size 3 μm, resolution: ~12 μm and 5-axis manipulator. Images thus obtained were qualitatively assessed for bone in-growth into the scaffolds.

### Statistical analysis

Radiological and histological images for all groups of animals were analyzed as means ± standard deviations and data has been analyzed by SPSS software package (Version 16, SPSS Inc., Chicago, USA) employing two-way ANOVA considering group and month as factors.

## Results and Discussions

### Bioactive glass powder characterization

**Figure 2:**
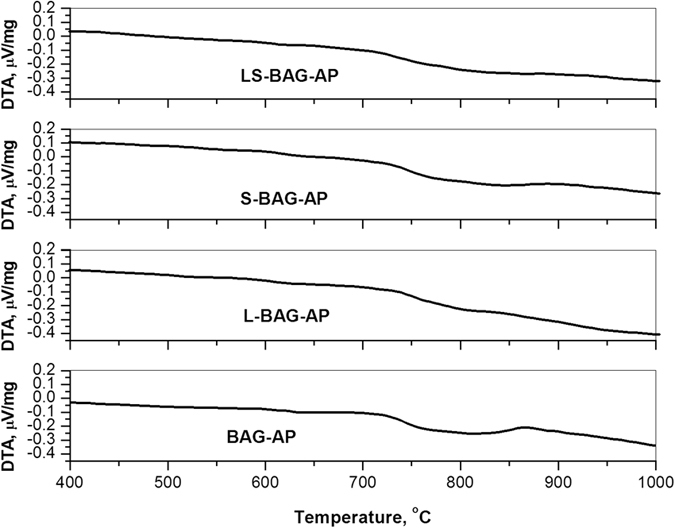
DTA profile of as-prepared samples for (**a**) BAG, (**b**) L-BAG, (**c**) S-BAG and (**d**) LS-BAG.

Fig. 2a–d shows the DTA thermogram of as-prepared powder samples (melted at 1450 °C) without and with Li/Sr doping. Glass transition temperatures (Tg) was found to be around 750 °C for BAG and 790, 780 and 770 °C respectively for L-BAG, S-BAG and LS-BAG with crystallization temperature 862 and 865 °C for BAG and L-BAG respectively. With the addition of dopants, Tg increases from the base composition (BAG) with associated enthalpy increase as well. No adsorbed or structural water loss noticed throughout the temperature regime. Heat treatment temperature of the porous green specimens fabricated later with this powders were selected based on the repeated trials on the porous green specimens with suitable strength, unaffected porous network inside and no incipience of glassy or crystalline phase.

**Figure 3:**
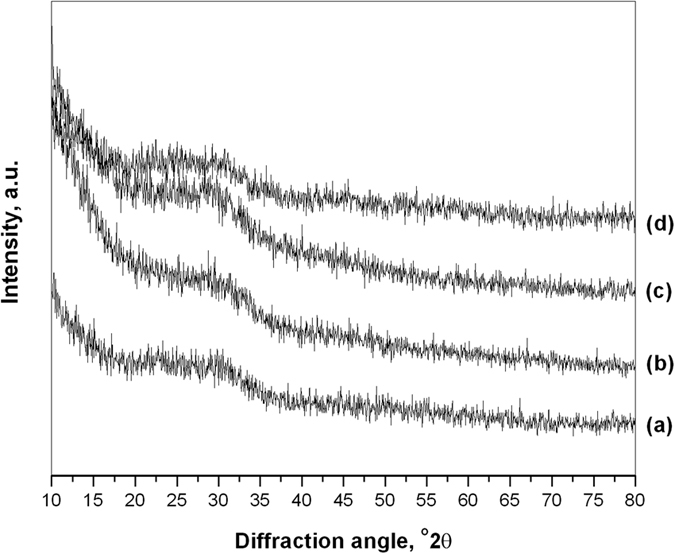
XRD patters of (**a**) BAG, (**b**) L-BAG, (**c**) S-BAG and (**d**) LS-BAG samples heat treated at their respective tempertaures.

XRD pattern (Fig. 3) of respective heat treated specimens for each composition confirms amorphous nature with broad diffraction at 2θ ranging between 20–35° indicative of disorder in the structure and glassy nature of powders. Addition of dopants had no appreciable influence on the glassy structure of base material except slight changes in amorphicity and no appearance of any crystalline peak.

**Figure 4:**
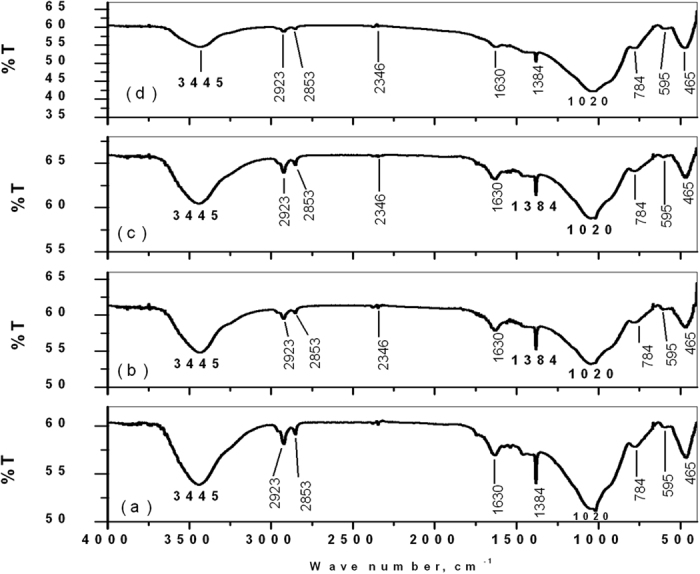
FTIR spectra of (**a**) BAG, (**b**) L-BAG, (**c**) S-BAG and (**d**) LS-BAG samples heat treated at their respective temperatures.

FTIR spectra (Fig. 4) of same powders show presence of hydroxyl (-OH) group around 3445 cm^−1^, along with Si-O-Si stretching frequency around 465 cm^−1^ and Si-O-Si bending frequency around 1020 cm^−1^ for all samples^31^. Other band assignments included Si-OH symmetric stretch at 780–980 cm^−1^ and vibrational mode of asymmetric stretch of Si-O-Si between 1100–1000 cm^−1^. The band assignments are summarized and are given in Table 3.

### Bioactive glass scaffolds characterzation

**Figure 5:**
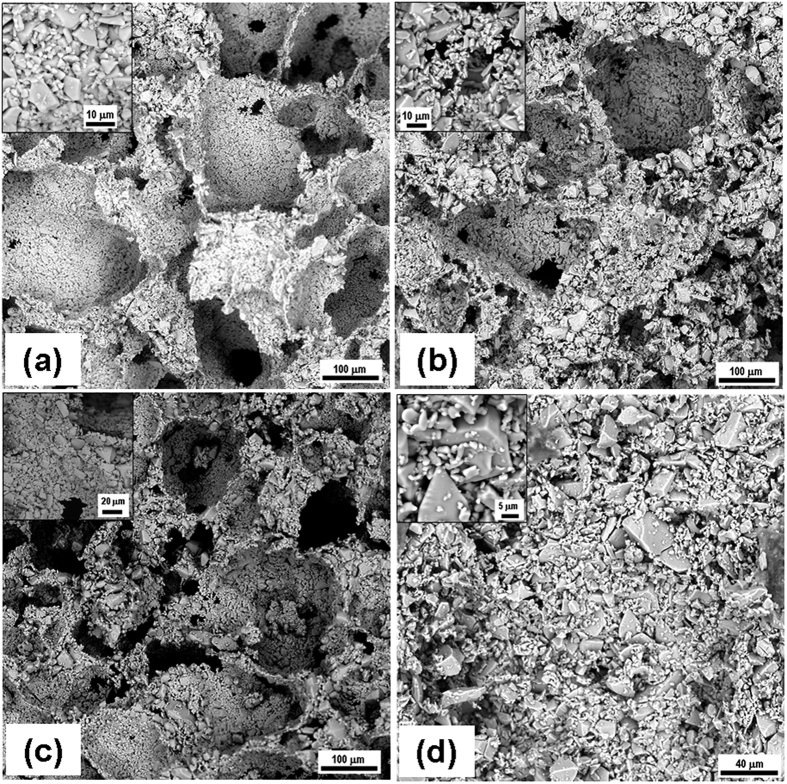
SEM microstructure of the porous scaffolds for all compositions (inset: higher magnified site).

Fig. 5a–d shows the SEM microstructure of the porous scaffolds for all compositions. Highly amorphous microstructures were obtained with presence of granular appearance throughout of the samples taken at different magnifications. A range of micro- to macro-pores were observed without any grains or crystals.

Due to the amorphous nature, green powders were fused at the boundary with presence of micro-pores between the fused powders. Mean pore sizes of BAG and L-BAG samples calculated by image processing was about 20 μm, while this was 47 μm and 8 μm for S-BAG and LS-BAG respectively. All the samples except LS-BAG showed presence of both micro- (10–50 μm) and macro-pores (>50 μm). For LS-BAG, it was more of coarsening than sintering of particles. For L-BAG, pore size was found to be in the range of 20–230 μm with 1–2 μm small pores throughout the microstructure. S-BAG on the other hand had pore size in the range of 30–260 μm with bi-modal distribution of pores in the range from 1–2 μm and 10–20 μm. Pore size range for LS-BAG was mainly in the range of 10–50 μm with presence of 1–2 μm of micro-pores. Amorphous content was found to be more in case of L-BAG and LS-BAG compared to S-BAG and BAG. Most probably Li had played a solute-drag effect for coarsening of the base glass particles. That means Li actually facilitated the coarsening so that green powder particles move against each other due to appearance of sharp melt at the interface. The effect was more evident in case of LS-BAG. Strontium on the other hand, did not have such effect as mentioned. As a consequence porous scaffolds made of BAG and S-BAG showed similar percentage of apparent porosity when heat treated as that of % naphthalene added while preparing the green compacts. But L-BAG and LS-BAG had much higher percentage of open porosity. As a result bulk densities of the samples were found to be higher in case of BAG and S-BAG than L-BAG and LS-BAG. The data (average values) are presented in Table 4.

### Simulated body fluid (SBF) study

**Figure 6:**
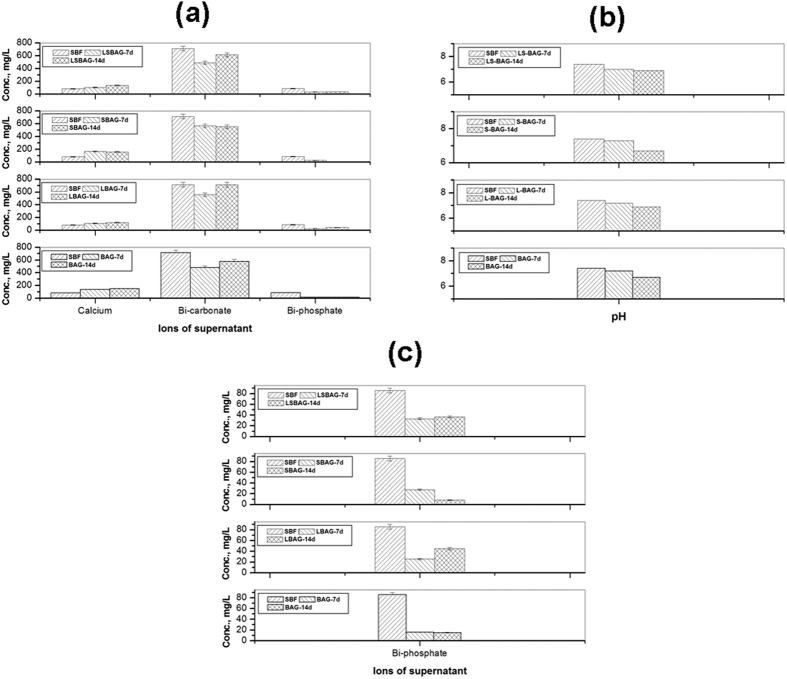
Variations of (**a**) concentration of supernatant (Ca^2+,^ HCO_3_^−^ and HPO_4_^2−^), (**b**) pH of SBF after days 7 and 14 in contact with the porous scaffolds (BAG, L-BAG, S-BAG and LS-BAG); (**c**) is the magnified part of HPO_4_^2-^ (**a**).

Fig. 6 shows a composite image showing variations of pH, concentrations of calcium, bi-carbonate and bi-phosphate in the supernatant with time, in contact with SBF. pH of the supernatant of all samples showed slight decreasing tendency with time and upto day 14, which corroborates our earlier findings on similar base glass^32^. For all the samples, Ca ion concentration of the supernatant was increased from 7 to 14 days except S-BAG, which showed increment of Ca ions at day 7 and continuous maintenance upto day 14. For BAG, L-BAG and LS-BAG this increase of Ca was due to dissolution from sample surface. HPO_4_^2−^ ion conc. on the other hand was decreased from pure SBF, most probably due to phosphate deposition on the surface. Carbonate in the supernatant, showed a decrement at day 7 and subsequent increment at day 14 which was possibly due to more carbonate deposition on the surface at day 7, more dissolution upto day 14 and eventually becoming saturated with the sample. The concentration of the supernatant analysis upto day 14 revealed bioactivity of the samples in terms of more and more -OH and PO_4_^3−^ ion deposition on the sample surface, which is a potential nucleation site for Ca after day 14 to form hydroxyapatite or carbonated apatite on its surface; but, S-BAG showed better bioactivity as the same deposition was prominent within day 14. The results obtained were compared with the MTT assay study shown later

**Figure 7:**
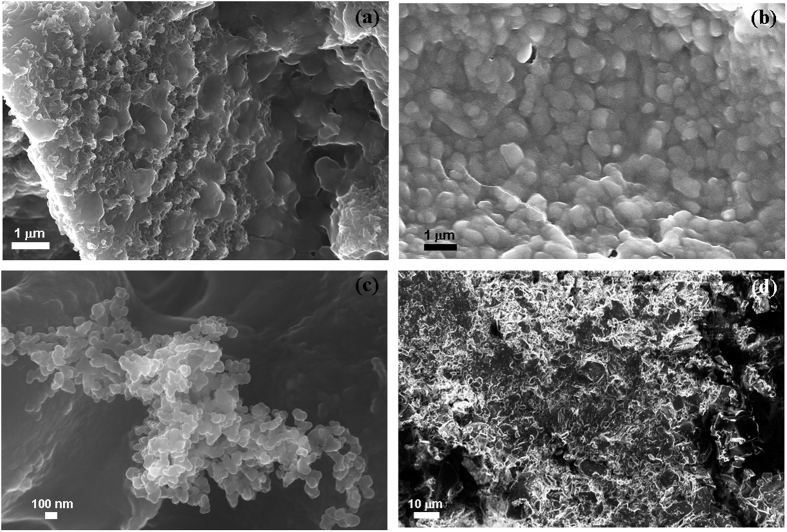
SEM microstructure of the porous scaffold surface after day 14 of SBF study; for (**a**) BAG, (**b**) L-BAG, (**c**) S-BAG and (**d**) LS-BAG.

Fig. 7 show the SEM microstructure of the porous scaffold surface after day 14 of SBF study. This shows formation of apatite like crystals on the surface of S-BAG (Fig. 7c) which was not very clear in case of other samples surfaces. L-BAG and LS-BAG showed amorphous nature of their respective surfaces (Fig. 7b,d), as kinetics of dynamic dissolution and deposition process of Ca^2+^, HCO_3_^−^ and HPO_4_^2−^ ions were still in continuation while for BAG (Fig. 7a), deposition of apatite like crystals have started.

### *In vitro* cell cyto-toxicity study

**Figure 8:**
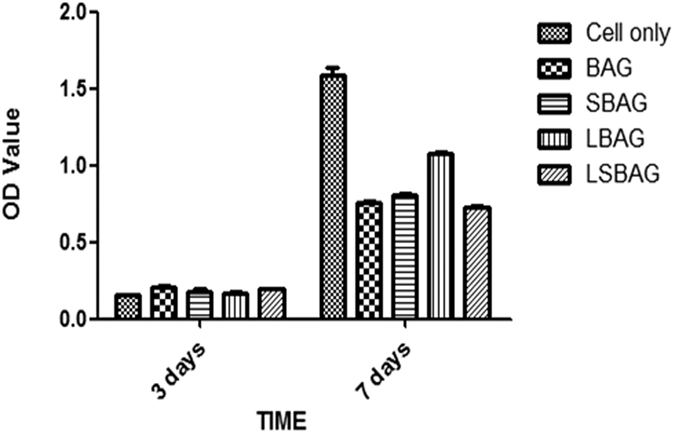
MTT assay results; calculated OD values for NIH3T3 expressed on the samples of BAG, L-BAG, S-BAG and LS-BAG after days 3 and 7.

From the calculated OD values (550 nm), percent cell (NIH3T3) proliferation was plotted against the days observed and is given in Fig. 8.

**Figure 9:**
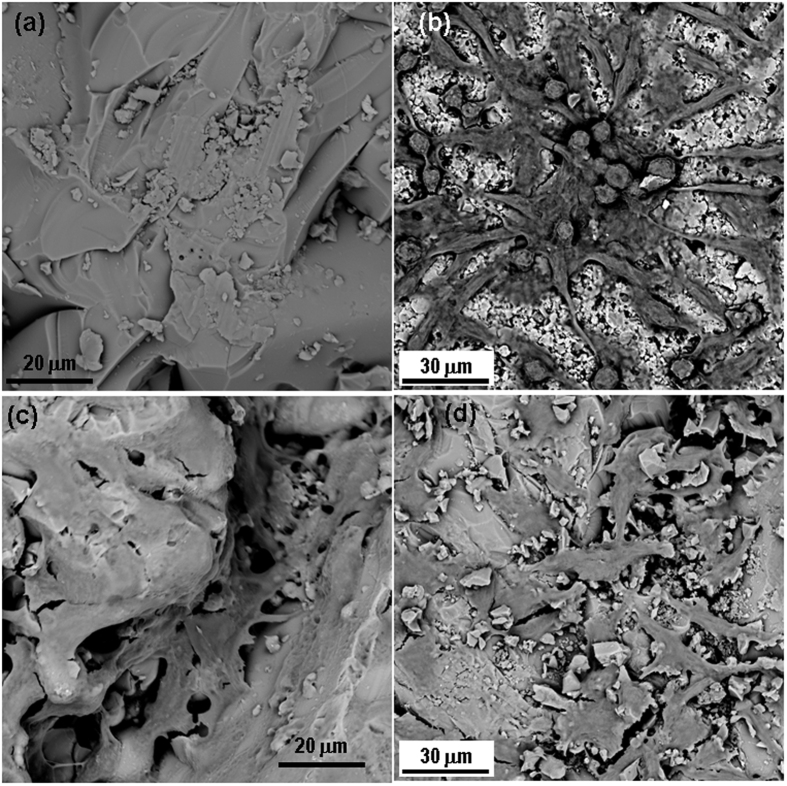
SEM cell morphology on the samples of (**a**) BAG, (**b**) L-BAG, (**c**) S-BAG and (**d**) LS-BAG after 7 days.

It was found that the initial proliferation of cells after day 3 was better than control due to initial attachment of cells. After 7 days, however, this was found to be better in case of L-BAG than the others. Cell growth rate was found to be reduced for all samples than control. All sample surface was considered as non-toxic and biocompatible. Cell morphology by SEM after day 7 was also revealed similar trend (Fig. 9) as L-BAG showed better NIH3T3 proliferation than other surfaces. Well-grown filopodia (microspikes) or cytoplasmic projections were seen and found to be more pronounced in this case. Filopodia contain actin filaments cross-linked into bundles by actin-binding proteins. Micropores present on the top of surface play pivotal role for better anchorage of the filopdia.

NIH3T3 mouse embryo fibroblast cell lines are regularly used for MTT assay to assess cyto-toxicity with respect to biomaterials’ effects on cell growth metabolism^33^. These cells have branched cytoplasm surrounding an elliptical nucleus and can be recognized by abundant rough endoplasmic reticulum and also synthesizes extracellular matrix (ECM) and collagen. NIH3T3 has also capability to detect substrate rigidity beyond the cell border^34^. From the results of Figs 8 and 9, it can be stated that there was substantial effect of Li alone to promote fibroblasts which was found to be least in case of BAG. S-BAG on the other hand, was not contributing extensively towards ECM formation and the gross effect of Li and Sr on cell proliferation was found to be least in case of LS-BAG (Fig. 8). S-BAG however showed better bioactivity in terms of apatite like crystal formation which will expected to contribute towards bone cell colonization *in vivo*. In this case, fibroblast cell extensions consolidated the pore site; ECM formed *in situ* alongwith HCO_3_^−/^/HPO_4_^2−^ deposition in contact with SBF after 7 days (Fig. 6) may help maturation of bone defect site faster *in vivo*. The combined effect of Li and Sr thus expected to be generate both soft and hard tisue *in vivo*. Most plausible reason behind slower rate of growth of NIH3T3 after 3 days may be the presence of other ions (e.g., Ca^2+^) which are also consistent with findings reported elsewhere stating that changes of extracellular calcium concentration can affect balance between proliferation and differentiation in fibroblasts. McNeil *et al.* demonstrated that elevation of extracellular calcium stimulates proliferation-associated signaling pathways in rat fibroblasts^35^.

### Bone in-growth evaluation by micro-CT

**Figure 10:**
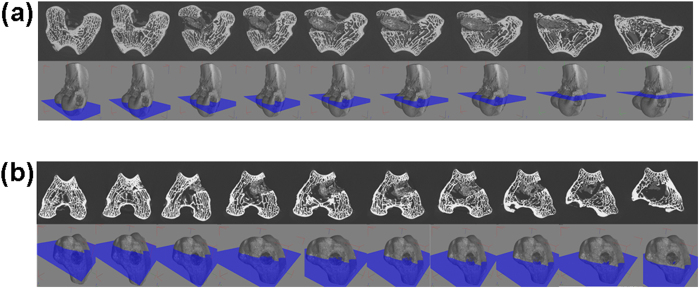
Serial slices along the Z-axis in micro-CT. Images are obtained for implanted BAG scaffolds. (**a**) After 2 months; (**b**) After 4 months.

Serial slices of X rays were carried out throughout Z-axis of a particular implanted bone section, images thus obtained were clubbed together and are given in Fig. 10a,b for BAG, 11a,b for L-BAG, 12a,b for S-BAG and 13a,b for LS-BAG after 2 and 4 months respectively. Serial images for BAG taken after 2 and 4 months showed that the porous scaffold has started degrading as revealed after 4 months but maintained its structure after 2 months. From the grey scale quantification it can be shown that BAG samples had higher amount of mature bone tissue after 4 months than the 2 months when more soft tissue apposition was evident. Stability of the implant thus impaired after 4 months which is anticipated to be continued and simultaneously converted to hard cortical tissue. Effect of lithium and strontium can be an interesting parameter which can dictate the degree of bone tissue conversion with time.

Serial sectional images of L-BAG after 2 months showed tissue invasion more in the central part of the implant than the periphery with a clear interfacial gap with the surrounding tissue. Grey scale values were found to be in between of cortical and cancellous tissues after 2 months which indicates that the implant was in the process of being resorbed which was continued up to 4 months. But the degree of resorption was higher in case of L-BAG than the BAG alone. L-BAG samples after 4 months showed very similar grey scale values with that of the cortical part of the bone. However, degradation of the samples was lowered for L-BAG than BAG alone.

S-BAG samples had clearly shown its efficacy expressed towards conversion to more of cortical tissue than the cancellous one. Cortical tissue could be seen at the periphery of the inserted samples as seen after 4 months. LS-BAG samples after 2 months showed close resemblance of the cancellous tissue on the periphery and cortical tissue in the inside of the implanted samples. After 4 months, the same phenomenon continued and the implanted samples were almost 60% converted to the surrounding bone. After 2 months, BAG samples only showed some interfacial gap which was absent for all doped samples.

3D images using micro CT are given in “Supplementary Information” as Fig. 1a,b for BAG, 2a and b for L-BAG, 3a and b for S-BAG and 4a and b for LS-BAG after 2 and 4 months respectively. The extent to which both soft and hard tissue apposed to BAG samples when implanted, cannot be assessed quantitatively from radiography. Tissue and blood vessels could be seen in the 3D representation of the micro CT. Porous nature both in and outside the medullary cavity were noticed in the 3D representation. Different grey scale values in the images represent quality of the bone and its degree of maturity. Implants could be seen from the 3D plots. It was found that mature bone tissue as well as blood vessels engulfed the implant which indicates good vascularisation. Porous nature of the cortical bone was observed in some sections. In a comparison, LS-BAG samples showed highest degree of tissue impregnation and vascularity among the four compositions. Strontium doping synergistically affected bone tissue apposition than glass without any doping. BAG scaffolds without doping actually had lower vascularity potential than the doped ones.

### Radiological examination

**Figure 14:**
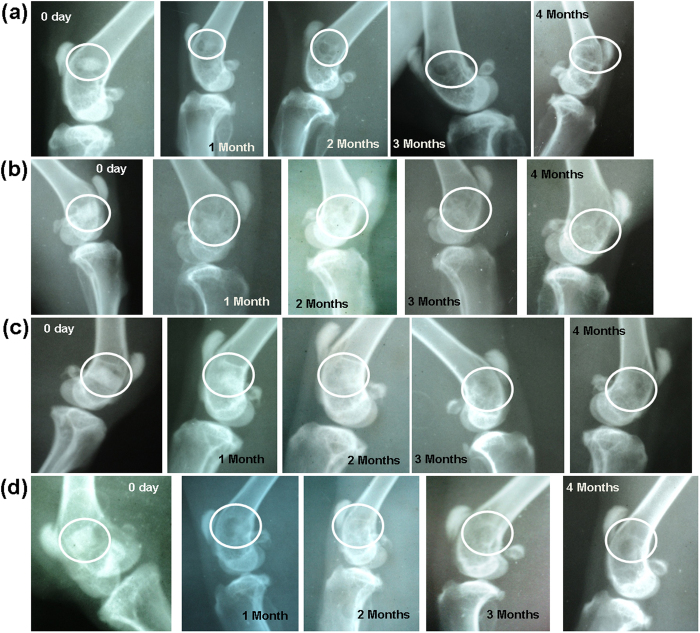
Radiographs taken at ‘0’ day, 1, 2, 3 and 4 months post-operatively implanted with (**a**) BAG, (**b**) L-BAG, (**c**) S-BAG and (**d**) LS-BAG.

Fig. 14a–d shows radiographs of defect surgery site and their interpretation is presented in Table 5. On the day of surgery, the distal metaphysis of femur showed presence of partial radiodense BAG implant (Fig. 14a) in the defect, which became moth-eaten on 1 month. The implant found to reduce in size gradually with implantation time and almost disappeared at 4 month with irregularly arranged bony tissue. In S-BAG samples (Fig. 14c), the defect radiodensity appeared to be unchanged after 1 month and the defect size shrunk at 2 months. At 3 months, only negligible amount of implant was present in the defect along with newly formed bony tissue and by the end of 4 months both the implant defects could be barely seen from the radiographs. L-BAG samples (Fig. 14b) also showed similar performance as that S-BAG, except that defect healing and new bone formation were enhanced. In LS-BAG samples (Fig. 14d), radiograph showed narrowing of bony defect and shrinkage of implant as early as one month post-operatively. Subsequent radiographs showed no traces of implant and defect. More importantly the radiodensity in defect area was almost identical to that of healthy bone.

### Histological evaluation

**Figure 15:**
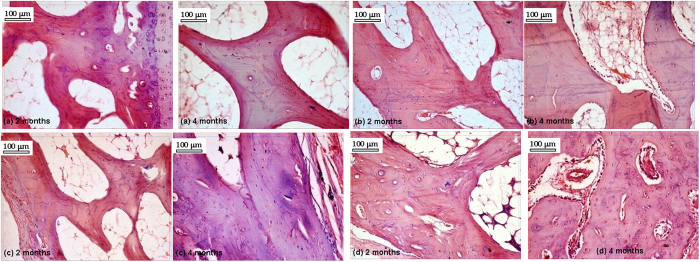
Histological sections taken after 2 and 4 months post-operatively implanted with (**a**) BAG, (**b**) S-BAG, (**c**) L-BAG and (**d**) LS-BAG.

Fig. 15 and Table 6 show the histological section images and evaluation report of bone-implant interface at 2 and 4 months after observing different cellular events. BAG scaffolds (Fig. 15a) showed well formed bony structure containing haversian system, canaliculi and sinusoidal spaces along with deposition of R.B.C., fat cells and scanty numbers of osteoblast in peri-medullary areas after 2 months. Strontium doped scaffolds (S-BAG: Fig. 15b) at 2 month showed prominent osteoblastic activity characterized by sufficient number of haversian canal, canaliculi, lacunae and osteoblastic cells with suitable cytoplasmic ratios. The bony matrix is invaded by highly proliferative branches of vessels containing sufficient amount of R.B.C, bony progenitor cells and focal calcified points. Similarly, L-BAG scaffolds (Fig. 15c) showed well developed bony structure with robust haversian system, osseous canaliculi and bony plates. The LS-BAG samples (Fig. 15d) depicted well formed osseous structure containing haversian canal, lamellae and canaliculi which was invaded by numerous blood vessels along with prominent osteoblastic and osteoclastic activities in the margin of lesion.

Histological evaluations at 4 months for the doped and pure bioactive glasses are also shown in Fig. 15a–d. Compared to 2 months, apparently higher angiogenesis was observed in all doped samples. However, angiogenic proliferation was more in LS-BAG and L-BAG samples compared to other samples. All doped samples showed highly proliferative stage of osteoblast and osteoclast cells (progenitor cells) along with foci of calcification.

### Fluorochrome labeling study

**Figure 16:**
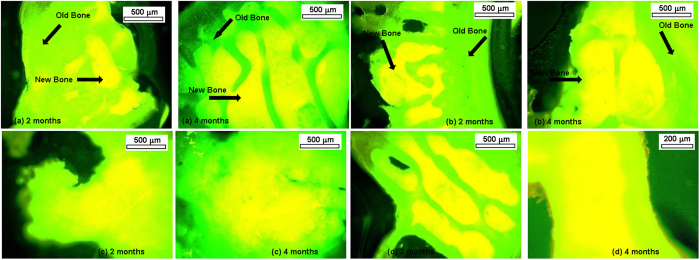
Fluorochrome labeling study (after oxytetracycline markings) taken after 2 and 4 months post-operatively implanted with (**a**) BAG, (**b**) S-BAG, (**c**) L-BAG and (**d**) LS-BAG.

Fig. 16 shows images of samples after oxytetracycline marking, where golden yellow florescence represents new bone and dark sea green indicates matured old bone. After 2 months, BAG scaffolds depicted double tone golden yellow fluorescence in a narrow zone in the defect site and the host bone looked dark sea green homogenous color. Relatively, better intensity of new bone formation (golden yellow fluorescence) was observed in S-BAG and LS-BAG at this time point. L-BAG at this time point also showed more new bone formation as compared to pure sample. At 4 months, all the samples depicted more new bone formation as compared to 2 month. However, distinct new bone formation was exhibited in all doped bioactive glass implants. LS-BAG implanted bone showed wide regions of golden yellow fluorescence (new bone formation) indicating rapid bone regeneration. S-BAG bone samples showed scattered and multiple regions new bone formation in defect area demonstrating their effectiveness in bone regeneration. Based on the calculation, percentage of bone formation through fluorochrome labeling images at two time point of 2 and 4 months have been done and is given in Table 7.

### Scanning electron microscopic (SEM) study

**Figure 17:**
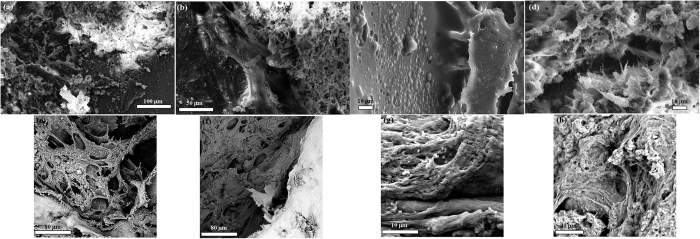
SEM images of bone-material (BAG, L-BAG, S-BAG and LS-BAG) interface taken after 2 months (**a–d**) and 4 months (**e–h**) post-operatively respectively.

Fig. 17 shows microstructural study of bone implant interface using SEM at two time point of 2 months (Fig. 17a–d) and 4 months (Fig. 17e–h). Effect of lithium and strontium could be established in SEM images and compared with the micro CT images. In all samples, with time, both soft and hard tissue got matured. L-BAG samples had a clear influence on soft tissue interaction with the sample (Fig. 17b,f). Collagenous network was prevalent in case of L-BAG while S-BAG samples (Fig. 17c,g) showed more of matured osteoblastic tissues apposed to the surface of the sample. LS-BAG on the other hand showed (Fig. 17d,h) both collagenous network and mature bone tissues, which was not only covered the surface but also had invaded the porous network structure of the implant. Interestingly, BAG samples after 4 months showed matured osteoblastic tissues (Fig. 17e), but there was also sporadic presence of RBCs on the surface of samples. Interfacial gap between the implanted samples and the surrounding bone was found more in case of BAG than the other samples. Bony networks could be seen for BAG but no collagenous microstructure. Interfacial gap was absent in case of L-BAG and LS-BAG after 4 months but like the BAG samples, S-BAG also revealed slight interfacial gap after 2 months which however completely absent after 4 months. Granular nature of the porous scaffolds revealed before animal experimentation were absent when implanted. There were no loose or unreacted glass particles after animal study.

Earlier *in vitro* and *in vivo* studies on porous bioactive glass scaffolds demonstrated their potentiality towards bone tissue engineering due to inherent osteoconductive and osteogenic properties^36^,^37^. Further improvement in biological properties of these scaffolds can be achieved via incorporation of suitable dopants that positively affect osteoblast activities thereby enable early new bone formation^6^. Amalgamation of trace metallic elements into tissue engineering constructs offer low cost, longer shelf life with low regulatory burden and low risk as compared to biologics. Due to these added benefits, delivery of trace metallic element as biological agents is getting considerable attention in tissue engineering and regenerative medicine applications^38^,^39^,^40^. To achieve toward this goal of improving the performance of bioactive glasses, a simpler method was identified to develop lithium and strontium doped glass and to investigate the mechanism of how these next-generation biomaterials can enhance both osteogenesis and angiogenesis for faster patient healing times and high surgical success rates.

Li^+^ is new additive ion of interest that brought attention due to its imminent role in osteogenesis. In a very recent study, Miguez-Pacheco *et al.* has shown that substitution of Na by different % of Li in 45S5 bioactive glasses cause decrease in T_g_ and T_m_ favoring sintering by viscous flow^41^ which also consolidates our present finding in case of L-BAG. However, we have also completed cell viability and proliferation studies to further strengthen its potential application. In another study, it has been reported that 75 patients treated with lithium were found to exhibit significantly greater bone mass in several areas compared to 75 normal participants^42^. Similarly, strontium (Sr^2+^), a non-essential element accounts for 0.035% of the calcium content in our skeleton system and has been shown to boost bone regeneration when incorporated into synthetic bone grafts^43^.

Li^+^ doping in bioactive glasses may inhibit GSK3, a negative regulator of the Wnt signaling pathway^44^. Moreover, it activates β-catenin-mediated T cell factor (TCF)-dependent transcription during bone and cartilage fracture healing^45^. Similarly, Sr^2+^enhances osteoclast apoptosis, increases pre-osteoblastic cell proliferation and collagen synthesis and thus decreases bone resorption and preserve bone formation^46^,^47^. In our study, radiological study revealed gradual replacement of scaffold with new bone after 2 months in all doped samples. Histological study revealed highly proliferative stage of osteoblast and osteoclast cells (progenitor cells) along with angio-proliferation component of bony tissue in doped samples compared to pure samples. This is presumably due to possible role of Sr and Li addition in bioactive glass. Sr^2+^ plays a vital role in overall bone turnover through early differentiation of osteoblast that helps in early expression of cbfa1 gene, indispensable for osteoblast differentiation^48^. Sr can also stimulate the calcium sensing receptor and other equivalent signaling pathways to induce early osteoblast differentiation^46^. The Wnt signaling pathway is one of the most key signal cascades in bone formation and remodeling process^49^,^50^. A direct link between BMP production and an activated Wnt signaling pathway in osteoblasts has been observed^51^,^52^. The activation of β-catenin signaling by Li+ shows its paramount role for fracture healing^45^. Higher osteoblastic activity and lamellar bone formation are prominent in binary Sr-Li doped bioactive glass which is due to combining effects of both dopants on bone formation processes of resorption and mineral aggregation.

Fluorochrome labeling using tetracycline marker is an indicator for the new bone formation, bone mineralization and remodeling^53^. These stains when incorporated will directly bind to areas undergoing calcification at the bone/osteoid (unmineralized bone) interface. After administration, tetracycline generally follows ionized calcium and deposited to the areas of mineralized tissue^54^,^55^. The labeled new bone and old bone emit bright golden-yellow and dark-sea green fluorescence respectively when observed under UV light. The method provides practical information in assessing the amount of new bone formation and bone healing^56^. In this study, at 2 months time point, the process of new bone formation was moderate in pure bioactive glass and relatively high in all three doped samples. In general, the activity of new bone formation was increased in all samples after 4 months. This may be due to the significant effects of single or binary dopants which may in turn help in cellular proliferation and osteoblastic activity. Previous studies established that Sr can influence cellular activities via the membrane bound calcium sensing receptor, both in osteoblasts and in cells of the osteoclasts lineage^15^,^57^,^58^. Moreover, Sr may enhance the ability of MSCs as well as pre-osteoblasts proliferation and differentiation into bone-forming osteoblasts^59^, through Wnt/b-catenin pathway by activating mitogenic signaling^60^. In a similar study using Sr doped HAp-based bioactive glasses implants, the sequential polychrome labeling of bone during *in vivo* osseointegration confirmed homogeneous bone formation around the test implants^61^.

SEM examination revealed both collagenous network and mature bone tissues in L-BAG while S-BAG samples showed more of matured osteoblastic tissues apposed to the surface of the sample. Interfacial gap between the implanted samples and the surrounding bone was found more in pure BAG than the doped other samples. Bony networks could be seen for pure BAG but no collagenous microstructure could be noticed, which is an indirect estimation of poorer bone quality in case of BAG than the other samples. Interestingly, sporadic presence of RBCs on the surface of samples indicating the healing was still continuing. Sr controls key proteolytic enzymes, matrix metalloproteinase-2 (MMP-2) and matrix metalloproteinase-9 (MMP-9) along with osteoprotegrin (OPG) and receptor activator of nuclear factor κ-β ligand (RANKL) that is produced by osteoblast cells and are key signaling mechanisms of osteoclast formation and its resorptive activity^62^,^63^. The combining effect of Sr enhances overall bone turn over by reduced osteoclastic resorption and an enhanced osteoblastic activity.

Micro CT in scaffold research has enabled accurate morphological studies to be carried out, yielding comprehensive data sets^64^,^65^. It also opened a new paradigm for investigations in tissue engineering^66^. Micro CT thus performed to understand the degree of vascularity as well as the interaction of soft and hard tissue with the material when implanted. In the present study, mature bone tissue as well as blood vessels engulfed the implant which indicates good vascularisation. In a comparison LS-BAG samples showed highest degree of tissue impregnation and vascularity among the four compositions probably due to the synergistic effect of lithium and strontium in particular. After 2 months, serial sectional images of L-BAG indicated that tissue invasion was more pronounced than in the central part of the implant than the periphery with a clear interfacial gap with the surrounding tissue. Grey scale values were found to be in between of cortical and cancellous tissues after 2 months which indicates that the implant was in the process of resorption. But the degree of resorption was higher in case of L-BAG than the BAG alone. S-BAG samples had clearly shown its efficacy expressed towards conversion to more of cortical tissue than the cancellous one. LS-BAG samples after 2 months showed close resemblance of the cancellous tissue on the periphery and cortical tissue in the inside of implanted samples. BAG samples only showed some interfacial gap which was absent for all doped samples. In a similar study, the bone regeneration ability of different bioactive glass particles has been observed in rabbit model^67^,^68^.

In summary, this study examines the effects of Sr and Li addition in bioactive glass compositions developed in the lab, the porous scaffolds made thereof for their physico-chemical, *in vitro* and *in vivo* osteogenetic properties alone and in combination. All bioactive glass both doped and undoped showed *in vivo* new bone formation during 4 months. Based on the microstructural, histological, radiological, micro CT and fluorochrome labeling results, Sr and/or Li doped bioactive glass showed acceleration of early-stage bone formation at the rabbit model defect site and Sr + Li doped bioactive glass proved to be more effective than other two compositions. Our findings suggest that incorporation of Sr and Li in bioactive glass can effectively enhance early stage *in vivo* osseointegration and bone remodeling properties. The results also suggest that doped bioactive glass might provide a delivery system for bioactive agents to accelerate bone healing and better anchorage of bone implants in orthopedic surgery. However, further detailed studies are needed to elucidate the exact mechanisms of Sr and Li dopants on enhanced bone healing for suitable use in future biomedical applications as a more osteoconductive bone substitute especially on earlier stages after implantation.

## Additional Information

**How to cite this article**: Khan, P. K. *et al.* Influence of single and binary doping of strontium and lithium on *in vivo* biological properties of bioactive glass scaffolds. *Sci. Rep.* **6**, 32964; doi: 10.1038/srep32964 (2016).

## Supplementary Material

Supplementary Information

## Figures and Tables

**Figure 11:**
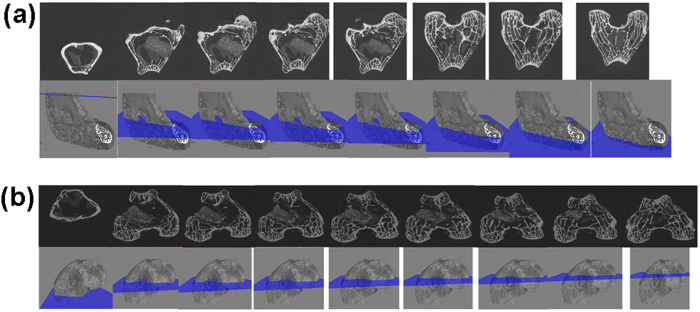
Serial slices along the Z-axis in micro-CT. Images are obtained for implanted L-BAG scaffolds. (**a**) After 2 months; (**b**) After 4 months.

**Figure 12:**
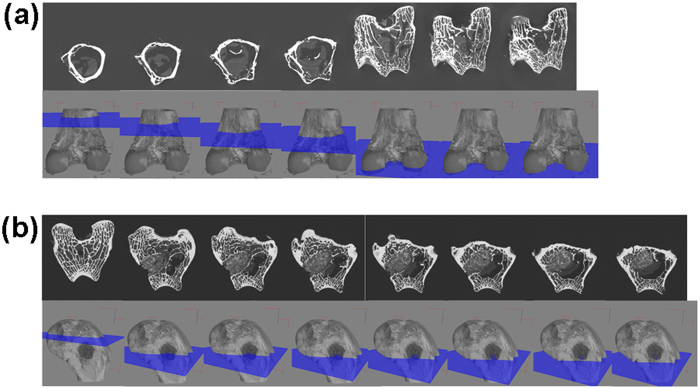
Serial slices along the Z-axis in micro-CT. Images are obtained for implanted S-BAG scaffolds. (**a**) After 2 months; (**b**) After 4 months.

**Figure 13:**
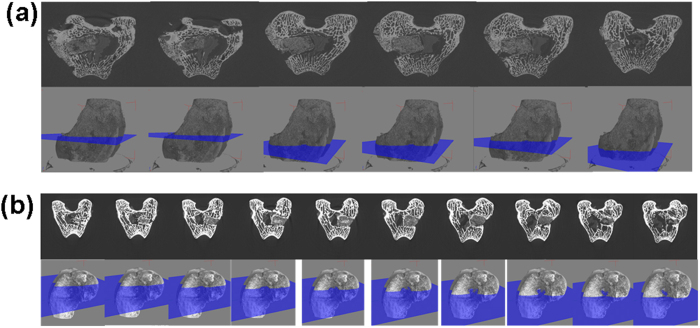
Serial slices along the Z-axis in micro-CT. Images are obtained for implanted LS-BAG scaffolds. (**a**) After 2 months; (**b**) After 4 months.

**Table 1:** Chemical composition of the as-prepared powders by ICP-AES.

Constituent	Mean wt. %			
	BAG	L-BAG	S-BAG	LS-BAG
SiO_2_	55.50	55.77	53.92	55.02
B_2_O_3_	1.38	1.76	2.33	1.00
CaO	23.60	23.95	23.00	23.36
Na_2_O	10.80	10.01	11.00	9.94
P_2_O_5_	5.74	5.85	5.59	5.59
TiO_2_	1.64	1.71	2.00	1.88
Li_2_O	—	0.22	—	0.30
SrO_2_	—	—	1.12	1.00

**Table 2:** Radiological scoring system (adopted as per Zhukauskas *et al.* 30).

Animal response	Score description
1	Bone just extending into the defect
2	Bone substantially bridging the cortical defect
3	Bone fully bridging the cortex without significant callus
4	Bone fully bridging the cortex with distinct overlying callus

**Table 3:** Band assignments for the peaks obtained for all samples (cf. Fig. 4).

Wave number (cm^−1^)	Band assignment	Wave number (cm^−1^)	Band assignment
465	δ (Si-O-Si) bending	1630	δ (OH)
595	P-O of PO_3_^2−^ group	2853	−OH (water)
784	ν (Si-O-Si) tetrahedral	2923	ν (CH)
1020	ν (Si-O-Si) asymmetric	3445	ν (OH)

**Table 4:** A.P. and B.D. data of the porous scaffolds.

Sample	A.P., %	B.D., g/c.c.
BAG	54.4 ± 1.63	1.16 ± 0.02
L-BAG	61.3 ± 1.84	0.97 ± 0.02
S-BAG	56.5 ± 1.7	1.09 ± 0.02
LS-BAG	64.5 ± 1.94	0.84 ± 0.02

**Table 5:** Radiological scoring values of different samples at different time intervals.

Group	1 month	2 month	3 month	4 month	SEM
S-BAG	0.66^a^	1.00^a^	1.66^b^	2.33^bc^	0.28
LS-BAG	0.66^a^	0.66^a^	1.33^b^	2.33^c^	0.28
LS-BAG	1.33^a^	1.66^ab^	2^bc^	3^c^	0.28
BAG	0.33^a^	0.66^b^	1^bc^	1.66^c^	0.28

Values are expressed as Mean ± SE. Values with different superscript within a row differs significantly (P < 0.001).

**Table 6:** Histological scoring values of different samples at 2 and 4 months.

Cellular Response	Time Point (2 month)				Time Point (4month)			
	Group 1	Group 2	Group 3	Group 4	Group 1	Group 2	Group 3	Group 4
Fibro vascular proliferation	1 ± 0.3	1.66 ± 0.3	1.33 ± 0.3	1.66 ± 0.3	1.33 ± 0.3	1.66 ± 0.3	1.33 ± 0.3	1.66 ± 0.3
Mononuclear cell	1 ± 0.23	1 ± 0.23	1 ± 0.23	1.33 ± 0.23	1 ± 0.23	1 ± 0.23	1.33 ± 0.23	1.33 ± 0.23
Osteoclast activity	1 ± 0.23	1.33 ± 0.23	1 ± 0.23	1.33 ± 0.23	1 ± 0.23	1 ± 0.23	1.33 ± 0.23	1.33 ± 0.23
Mucin deposit	1.33 ± 0.16	1 ± 0.16	1 ± 0.16	1.33 ± 0.16	1 ± 0.16	1 ± 0.16	1 ± 0.16	1 ± 0.16
Vascularisation	1 ± 0.26	1.33 ± 0.26	1 ± 0.26	1.66 ± 0.26	1.33 ± 0.26	1.66 ± 0.26	1.33 ± 0.26	2 ± 0.26
Osteoblastic activity	1 ± 0.31	1.66 ± 0.31	1.33 ± 0.31	1 ± 0.31	1.33 ± 0.31	1.66 ± 0.31	1.66 ± 0.31	2.3 ± 0.31

Values are expressed as Mean ± SE.

**Table 7:** Percentage of bone formation through fluorochrome labeling images at two time point of 2 and 4 months a, b, c, d means with different superscripts within a column differs significantly among the treatment (p < 0.01).

Treatment	2 months	4 months
BAG	32.47 ± 0.439^bA^	48.29 ± 0.541^bB^
BAG-Sr	23.663 ± 0.513^aA^	45.432 ± 0.573^aB^
BAG-Li	39.459 ± 0.562^cA^	51.466 ± 0.584^cB^
BAG-Sr + Li	47.459 ± 0.513^dA^	54.897 ± 0.588^dB^

A, B mean with different superscript within a row differs significantly between the month of sample within a treatment (p < 0.001).
