# Supplementary material for: Influence of single and binary doping of strontium and lithium on in vivo biological properties of bioactive glass scaffolds
Source: Sci Rep. 2016 Sep 8;6:32964. doi: 10.1038/srep32964 (PMC5015095; doi:10.1038/srep32964)
Supplement: Supplementary Information [file srep32964-s1.doc]

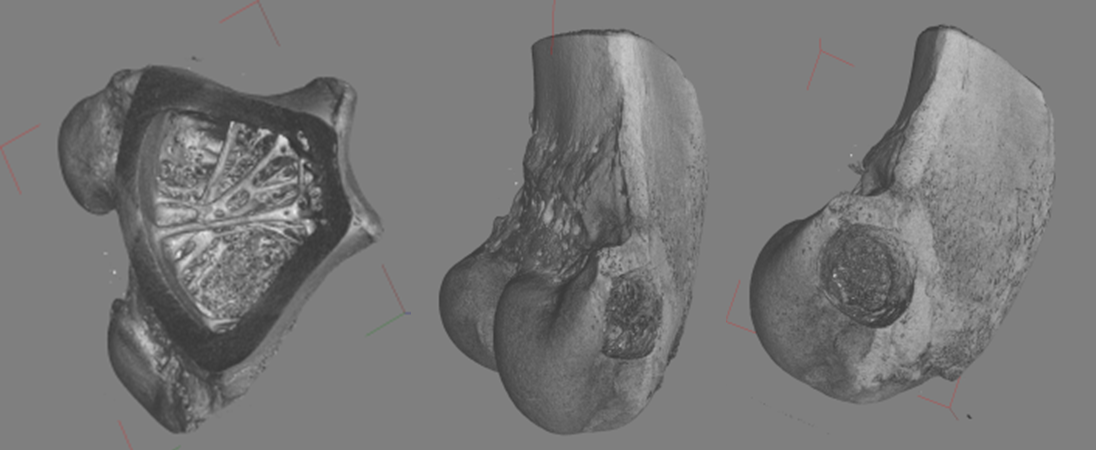


(a)


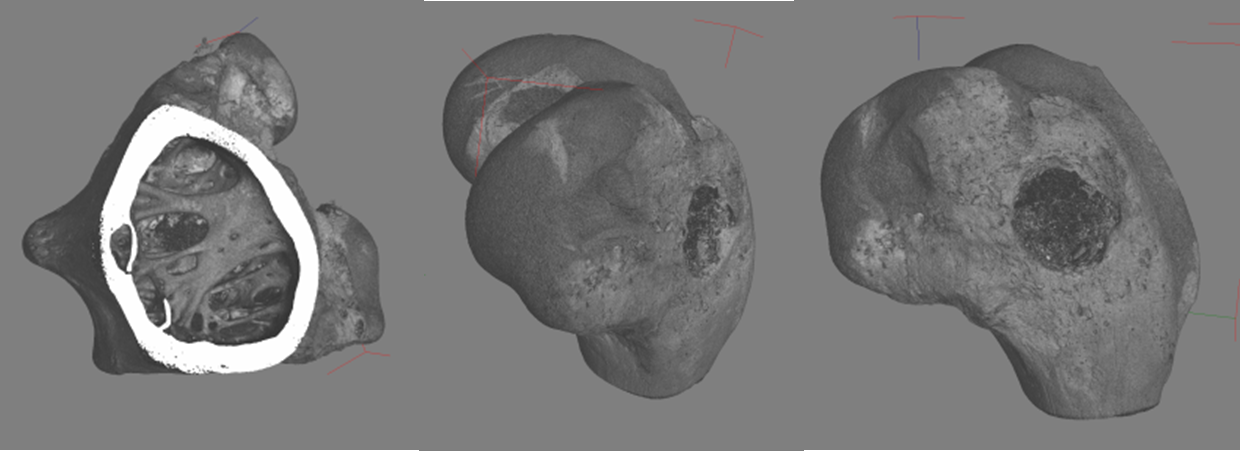


(b)

Fig. 1: 3D images using micro CT for BAG sample after (a) 2 and (b) 4 months


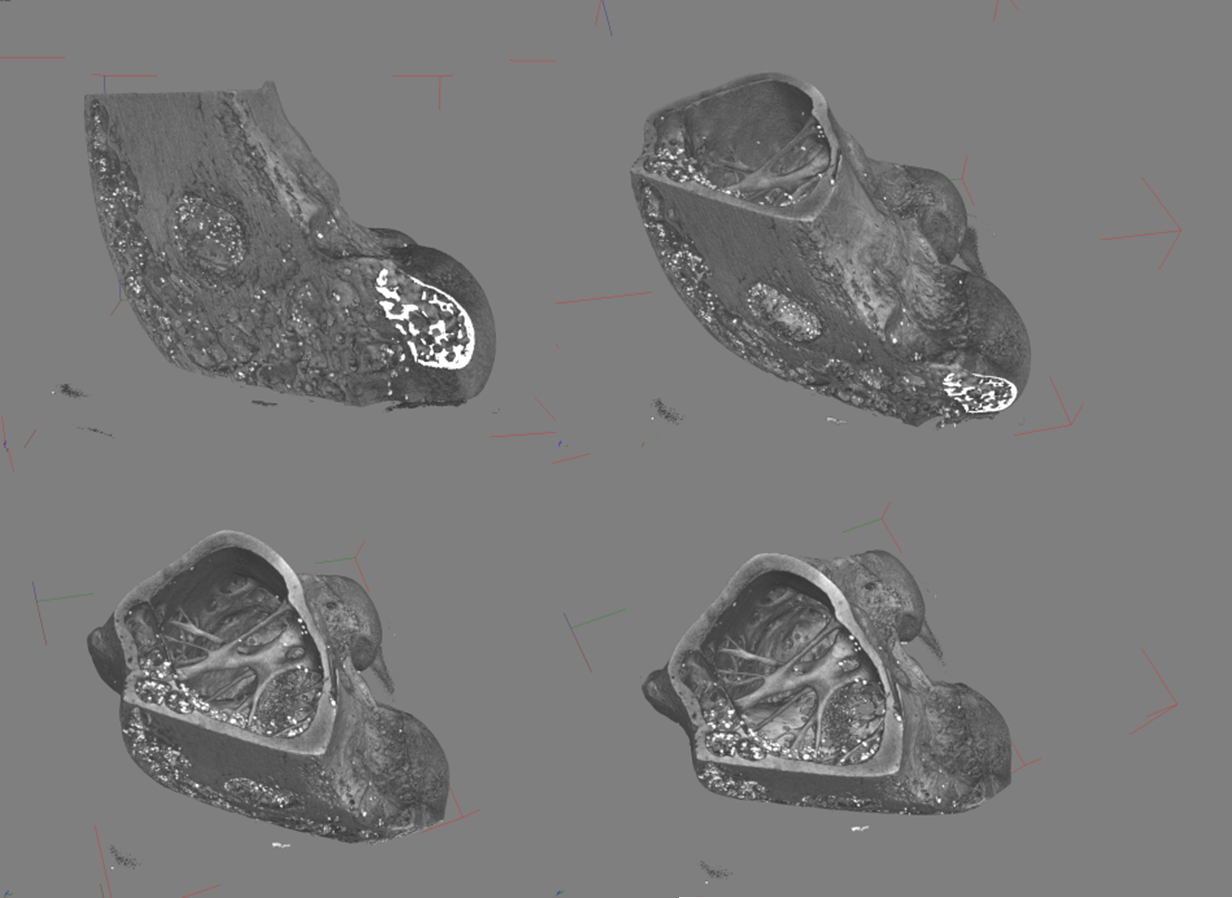


(a)


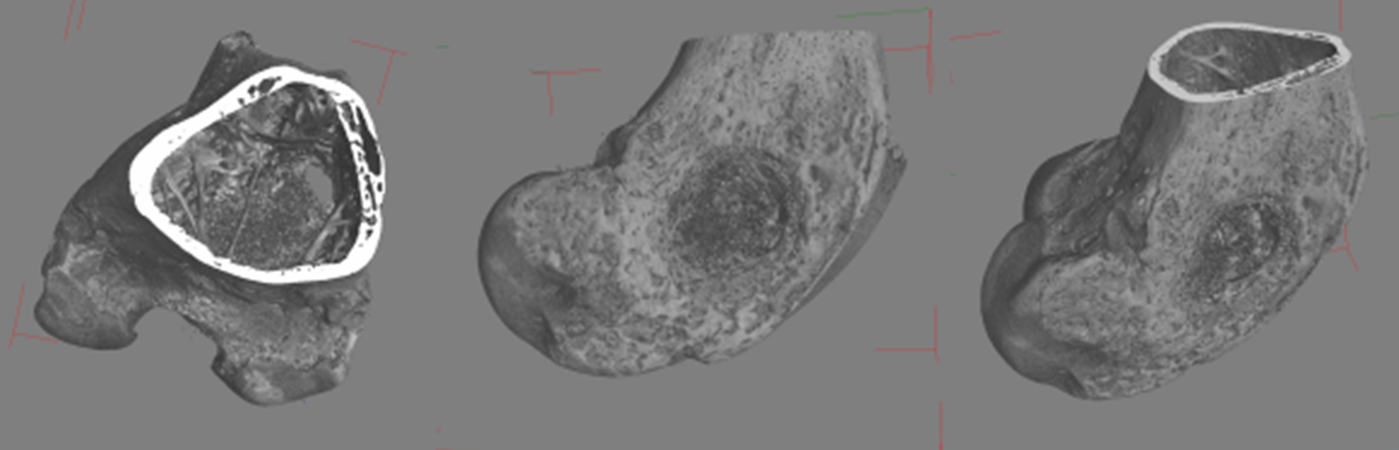


(b)

Fig. 2: 3D images using micro CT for L-BAG sample after (a) 2 and (b) 4 months


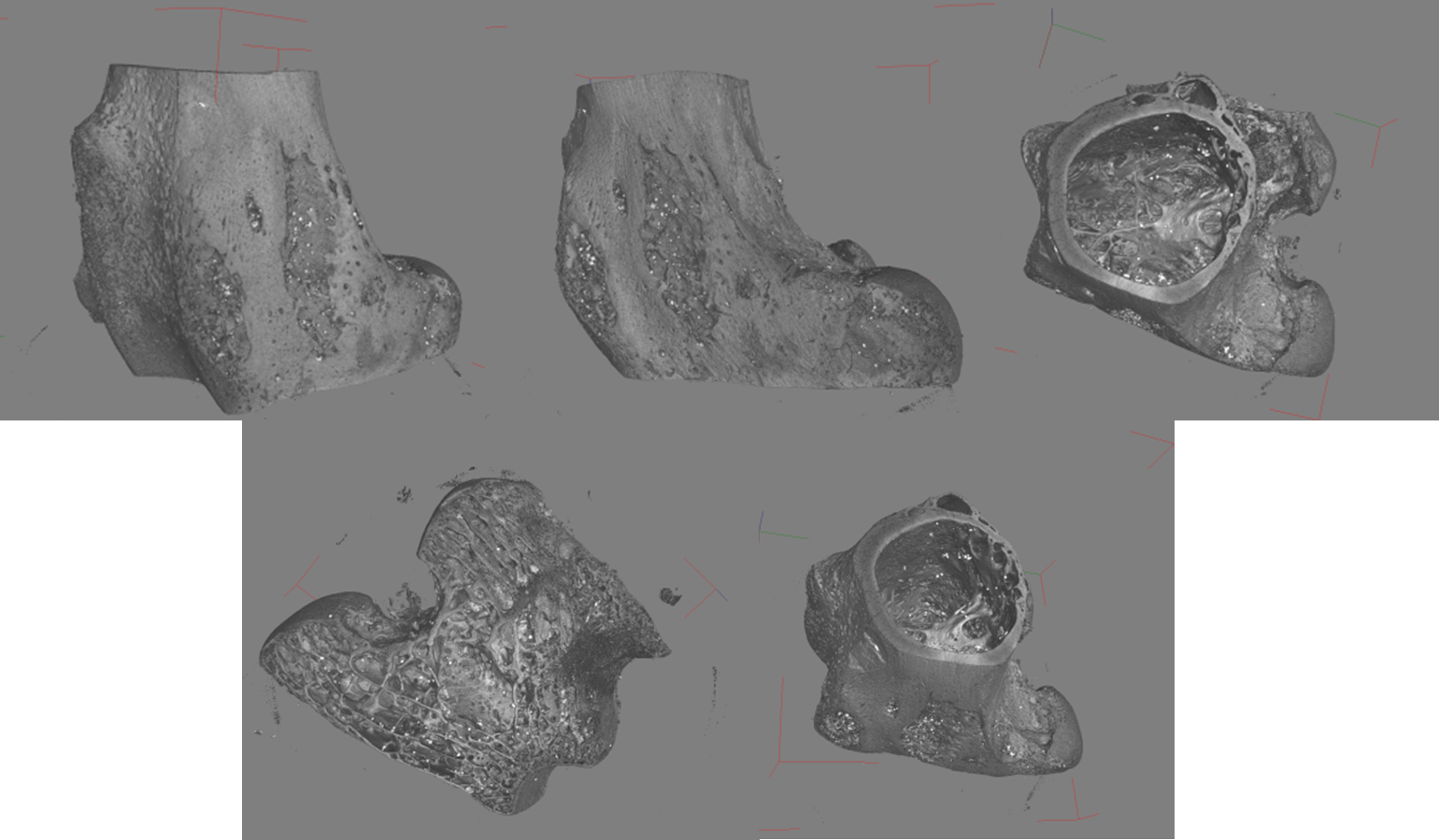


(a)


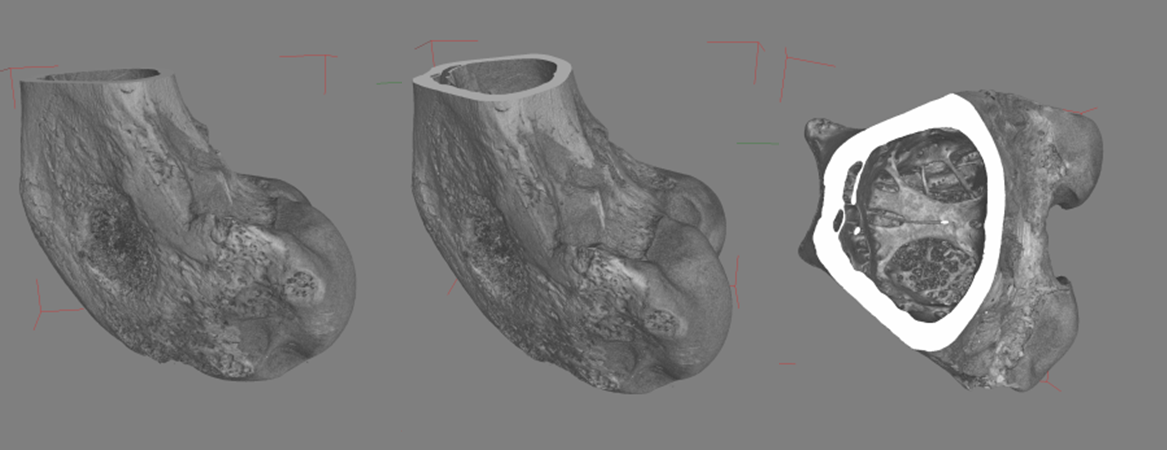


(b)

Fig. 3: 3D images using micro CT for S-BAG sample after (a) 2 and (b) 4 months


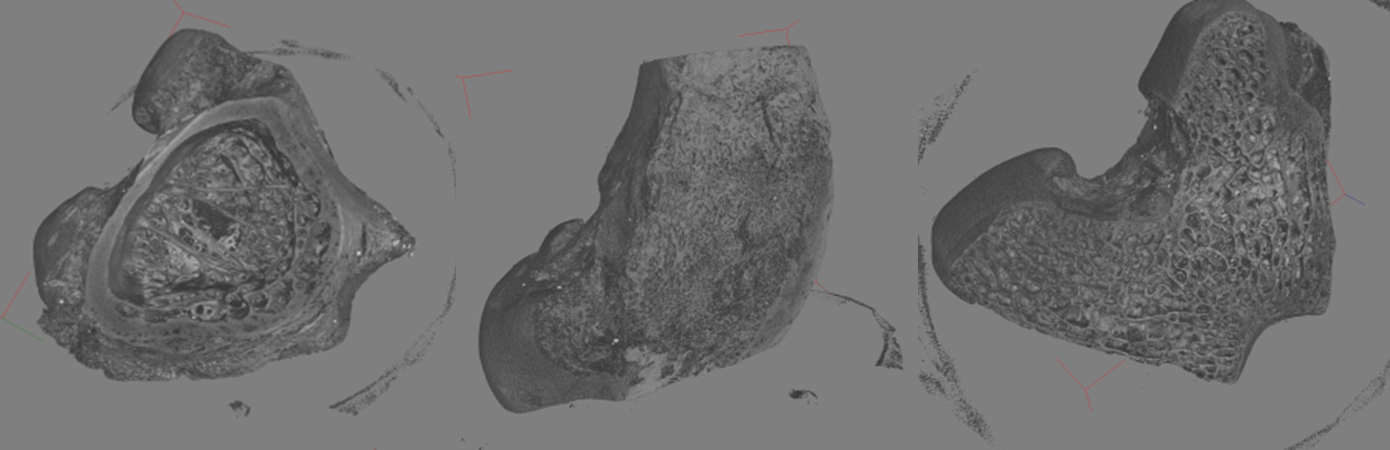


(a)


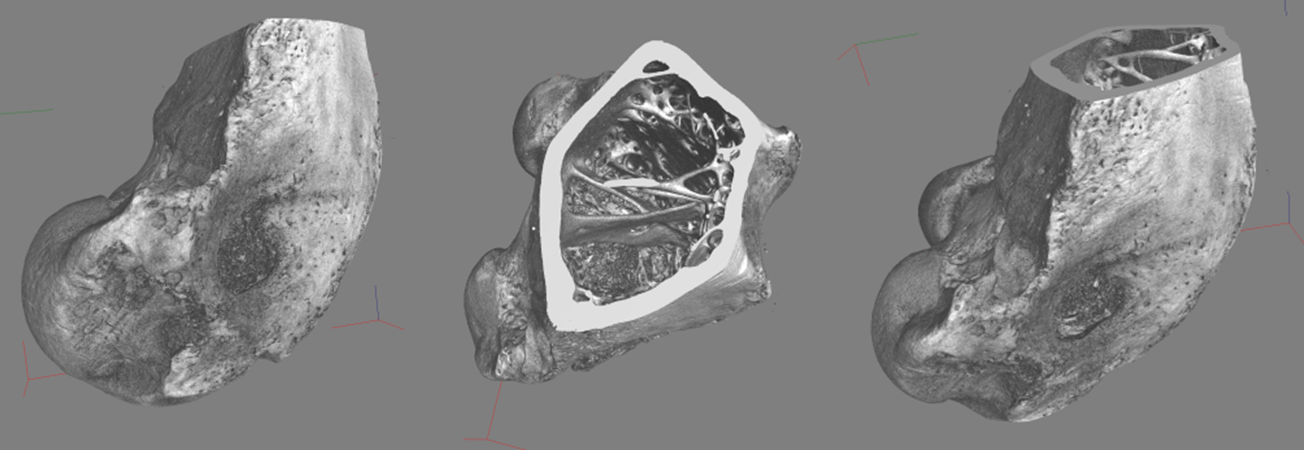


(b)

Fig. 4: 3D images using micro CT for LS-BAG sample after (a) 2 and (b) 4 months
